# Quercetin Attenuates Manganese-Induced Neuroinflammation by Alleviating Oxidative Stress through Regulation of Apoptosis, iNOS/NF-κB and HO-1/Nrf2 Pathways

**DOI:** 10.3390/ijms18091989

**Published:** 2017-09-15

**Authors:** Entaz Bahar, Ji-Ye Kim, Hyonok Yoon

**Affiliations:** 1 College of Pharmacy, Research Institute of Pharmaceutical Sciences, Gyeongsang National University, Jinju 52828, Gyeongnam, Korea; entaz_bahar@yahoo.com; 2 Department of Pathology, College of Medicine, Yonsei University, Seoul 03722, Korea; alucion@gmail.com; 3 Department of Pathology and Translational Genomics, School of Medicine, Samsung Medical Center, Seoul 06351, Korea

**Keywords:** manganese, manganism, quercetin, oxidative stress, neuroinflammation, apoptosis

## Abstract

Manganese (Mn) is an essential trace element required for the development of human body and acts as an enzyme co-factor or activator for various reactions of metabolism. While essential in trace amounts, excessive Mn exposure can result in toxic accumulations in human brain tissue and resulting extrapyramidal symptoms called manganism similar to idiopathic Parkinson’s disease (PD). Quercetin (QCT) has been demonstrated to play an important role in altering the progression of neurodegenerative diseases by protecting against oxidative stress. This study aimed to investigate the protective effect of QCT on Mn-induced neurotoxicity and the underlying mechanism in SK-N-MC human neuroblastoma cell line and Sprague-Dawley (SD) male rat brain. The results showed that Mn treatment significantly decreased the cell viability of SK-N-MC cell and increased the release of lactate dehydrogenase (LDH), which was attenuated by QCT pretreatment at 10 and 20 µg/mL. Compared to the Mn alone group, QCT pretreatment significantly attenuated Mn-induced oxidative stress, mitochondrial dysfunction and apoptosis. Meanwhile, QCT pretreatment markedly downregulated the NF-κB but upregulated the heme oxygenase-1 (HO-1) and Nrf2 proteins, compared to the Mn alone group. Our result showed the beneficial effect of QCT on hematological parameters against Mn in rat brain. QCT decrease reactive oxygen species (ROS) and protein carbonyl levels and increased Cu/Zn-superoxide dismutase (SOD) activity induced in Mn-treated rats. QCT administration caused a significant reduction in the Mn-induced neuroinflammation by inhibiting the expression of inflammatory markers such as tumor necrosis factor-α (TNF-α), interleukin-1β (IL-1β), interleukin-6 (IL-6) cyclooxygenase-2 (COX-2) and inducible nitric oxide synthase (iNOS). QCT lowered the Mn elevated levels of various downstream apoptotic markers, including Bax, cytochrome *c*, cleaved caspase-3 and polymerase-1 (PARP-1), while QCT treatment upregulated anti-apoptotic Bcl-2 proteins and prevented Mn-induced neurodegeneration. Furthermore, administration of QCT (25 and 50 mg/kg) to Mn-exposed rats showed improvement of histopathological alteration in comparison to Mn-treated rats. Moreover, administration of QCT to Mn-exposed rats showed significant reduction of 8-hydroxy-2′-deoxyguanosine (8-OHdG), Bax, activated caspase-3 and PARP-1 immunoreactivity. These results indicate that QCT could effectively inhibit Mn induced apoptosis and inflammatory response in SK-N-MC cells and SD rats, which may involve the activation of HO-1/Nrf2 and inhibition of NF-κB pathway.

## 1. Introduction

Manganese (Mn) is an abundant and essential trace element, especially critical for normal development and function of human body [1]. Mn is required as an enzyme co-factor or activator for various metabolic reactions, i.e., Mn binds to and/or regulates several important body enzymes such as Mn-superoxide dismutase (Mn-SOD) and pyruvate carboxylase that play important role in the growth and development of the central nervous system (CNS) [2].

Manganism, similar to idiopathic Parkinson’s disease (PD) is a CNS disorder caused by toxic exposure to manganese (Mn) characterized by a broad spectrum of neurological deficits including locura manganica, motional lability, compulsive behavior and visual hallucinations [3,4,5,6]. Manganism has been reported through occupational (e.g., welder, smelters), environmental (fuel additive methylcyclopentadienyl manganese tricarbonyl), medical (liver diseases, prolonged parenteral nutrition and abuse of illicit drugs), and dietary overexposure of Mn [7,8,9,10,11,12,13,14]. Several studies have reported some different aspects of manganism, and proposed the possible underlying mechanisms but it still not well understood. It has been reported, mitochondria as the primary target organelle for manganism [15,16]. Mn can induce reactive oxygen species (ROS) generation which significantly affects mitochondrial function, impairs endoplasmic reticulum (ER) hemostasis and promote apoptosis [11,17,18,19].

Quercetin (QCT) (3,3′,4′,5,7-pentahydroxyflavone), an important naturally occurring dietary polyphenol present in red onions, apples, berries, citrus fruits, tea, and red wine [20,21,22], has been reported to possess strong anti-oxidant, anti-inflammatory activity that can prevent many diseases including, neurodegenerative disorder, diabetes, obesity and cancer [21,23,24,25,26]. QCT plays an important role in altering the progression of neurodegenerative diseases by its protective effect against oxidative stress [27]. QCT blocked both the cyclooxygenase and lipooxygenase pathways at relatively high concentrations, while at lower concentrations; the lipooxygenase pathway was the primary target of inhibitory anti-inflammatory activity [28]. Previous study suggested that pretreatment of primary neuron cell cultures with quercetin significantly attenuated amyloid β-induced cytotoxicity, protein oxidation, lipid-peroxidation, and apoptosis [29]. Treatment with QCT caused a decrease in the carbonyl content in erythrocytes subjected to oxidative stress [30]. Recent reports showed that quercetin may exert its protective action against inflammation through inhibition of inducible nitric oxide synthase (iNOS)/NF-κB and induction of Nrf2/heme oxygenase-1 (HO-1) pathways [31,32,33,34]. Studies also indicated the potential benefit of QCT treatment for conditions involving mitochondrial dysfunction associated with increased oxidative stress [35].

In the present study, we have investigated the protective effects of QCT against Mn-induced neuroinflammation and the underlying mechanism, including the mechanistic study of Mn neurotoxicity in dopaminergic SK-N-MC human neuroblastoma cells and Sprague-Dawley (SD) male rats.

## 2. Results

### 2.1. Protective Effect of QCT on Mn-Induced Cytotoxicity in SK-N-MC Cells

**Figure 1:**
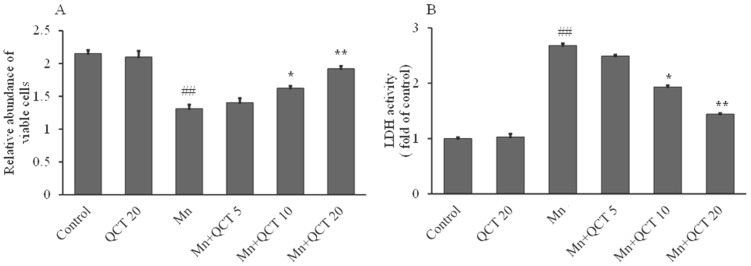
Protective effect of Quercetin (QCT) on Mn-induced cell cytotoxicity in SK-N-MC cell lines. (**A**) Cell viability; (**B**) lactate dehydrogenase (LDH) activity. Values were represented as mean ± SD. ^##^ *p* < 0.001 as compared with the control group; * *p* < 0.01 ** *p* < 0.001 as compared with the Mn alone group.

The effect of QCT on the viability of SK-N-MC cells cultured under Mn-induced toxicity conditions was measured by crystal violet assay. Pretreatment of SK-N-MC cells with QCT at concentrations of 5, 10 and 20 µg/mL protected SK-N-MC cells from Mn toxicity. An increase in cell viability was observed in QCT-treated cells compared to Mn alone group (Figure 1A). The result displayed that QCT (10 and 20 μg/mL) possessed the best protective effects. Correspondingly, QCT pretreatment markedly decreased (*p* < 0.01 or *p* < 0.001) the Mn-caused lactate dehydrogenase (LDH) release (Figure 1B). QCT alone treatment did not change the cell viability and LDH activity, compared to the control group (Figure 1).

### 2.2. QCT Attenuated Mn-Induced Oxidative Stress in SK-N-MC Cells

**Figure 2:**
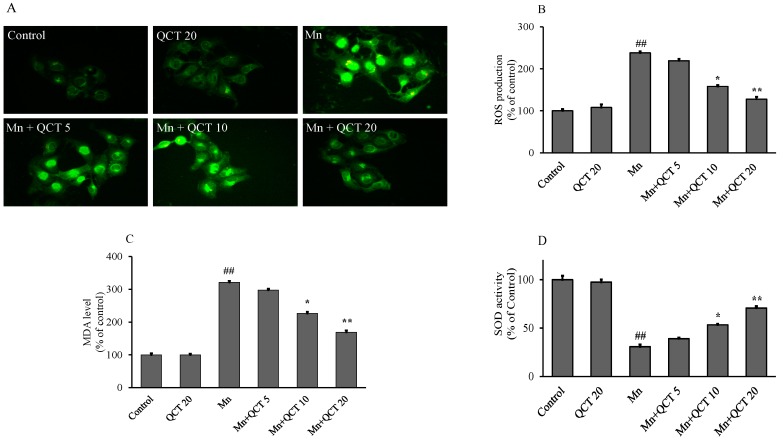

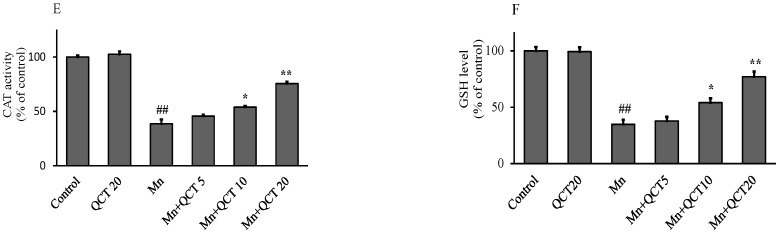
Protective effect of QCT on Mn-induced oxidative stress in SK-N-MC cell lines. (**A**) Morphologic images of intracellular ROS generation using 2′,7′-dichlorofluorescein diacetate (DCHF-DA) staining, scale bars: 100 µm ; (**B**) ROS generated relative to control were quantified; (**C**–**F**) impact of QCT treatment on cellular malondialdehyde (MDA), superoxide dismutase (SOD), catalase (CAT), and glutathione (GSH) levels, respectively. Values were represented as mean ± SD. ^##^ *p* < 0.001 as compared with the control group; * *p* < 0.01 and ** *p* < 0.001 as compared with the Mn alone group.

As shown in Figure 2A, B after exposed to 500 μM Mn for 24 h, the intracellular ROS level of the SK-N-MC cells markedly increased to 238% (*p* < 0.001) relative to the control. When the cells were pretreated with different concentrations of QCT (5, 10 and 20 μg/mL) in the presence of 500 μM Mn for 24 h, the intracellular ROS levels significantly decreased to 206, 159 (*p* < 0.01), and 125% (*p* < 0.001) of the control value, respectively. Similarly, the cells were pretreated with different concentrations of QCT (5, 10 and 20 μg/mL) in the presence of Mn (500 μM) for 24 h significantly decreased (*p* < 0.001) the malondialdehyde (MDA) levels from 321.084% to 297.59%, 226.50% (*p* < 0.01) and 168.67% (*p* < 0.01) (Figure 2C), respectively. Correspondingly, QCT pretreatment at 10 and 20 μg/mL markedly increased the activities of SOD and catalase (CAT) and the intracellular levels of glutathione (GSH) (*p* < 0.01 or *p* < 0.001) (Figure 2D–F). QCT alone treatment at 5, 10 and 20 μg/mL had no effect on cellular oxidative stress.

### 2.3. QCT Attenuates Mn-Induced the Loss of Mitochondrial Membrane Potential (ΔΨm) and Apoptosis in SK-N-MC Cells

**Figure 3:**
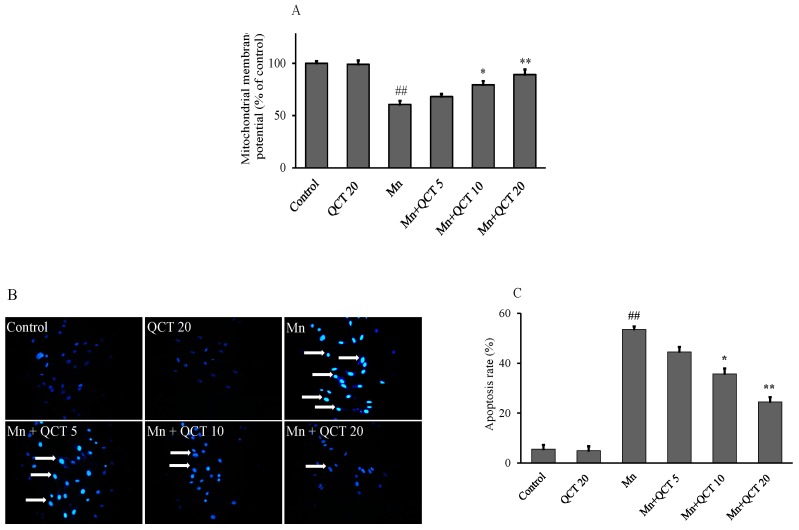
Protective effects of QCT on the cell survival in Mn-treated SK-N-MC cells. (**A**) Mitochondrial membrane potential (ΔΨm) determined by monomeric JC-1 green fluorescence emission and aggregated method; (**B**) representative images by Hoechst 33342 staining, arrowheads in the pictures indicate the nuclei of the apoptotic cells (Hoechst-positive cells), scale bar: 100 µm ; (**C**) the apoptosis rate was determined by calculating the percentage of Hoechst positive cells over the total number of cells. Values were represented as mean ± SD. ^##^ *p* < 0.001, compared to the control group; * *p* < 0.01, ** *p* < 0.001, compared to the Mn alone group.

The changes of ΔΨm were examined and analyzed using a sensitive fluorescent dye 5,5′,6,6′-Tetrachloro-1,1′,3,3′-tetraethylbenzimidazolylcarbocyanine iodide (JC-1). As shown in Figure 3A, Mn exposure significantly decreased (*p* < 0.001) the fluorescence density of JC-1 in SK-N-MC cells, indicating Mn caused loss of ΔΨm. Compared to the control, the Mn-treated alone group showed a decreased ΔΨm at 60.60%, which could be reverted to 68.19%, 79.43% (*p* < 0.01) and 89.27% (*p* < 0.001) of the control value by QCT pretreatment at the final concentrations of 5, 10 and 20 μg/mL, respectively. QCT alone treatment did not affect the level of ΔΨm (Figure 3A). SK-N-MC cells treated with 500 μM Mn for 24 h showed typical characteristics of apoptosis, including the condensation of chromatin, the shrinkage of nuclei using Hoechst 33342 staining as shown in (Figure 3B). The amount of apoptotic nuclei was markedly increased, and the apoptotic rate was significantly increased (*p* < 0.001) relative to the control group. However, the number of apoptotic cell was significantly decreased (*p* < 0.01 or *p* < 0.001) with QCT pretreatment at 10 and 20 μg/mL in the presence of Mn (Figure 3C).

### 2.4. QCT Down-Regulates the P-IκBα/NF-kB and Up-Regulates the HO-1/ Nrf2 Activity in SK-N-MC Cells

**Figure 4:**
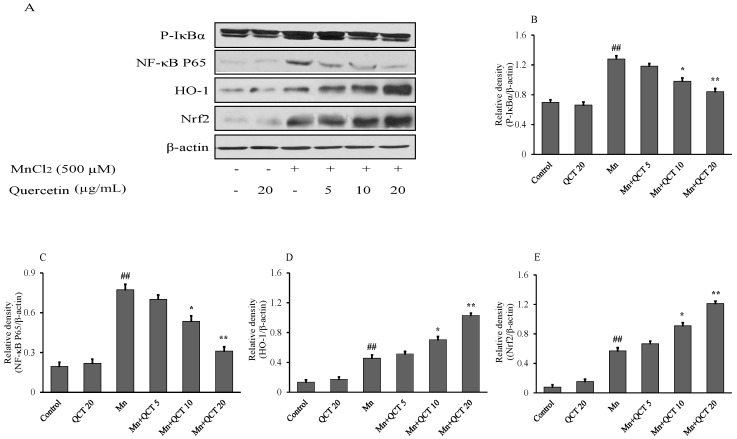
Effect of QCT on Mn-induced P-IκBα, NF-κB P65, Nrf2 and HO-1 activity in SK-N-MC cells. (**A**) The western blot analysis along with (**B**–**E**) relative density histograms of the P-IκBα, NF-κB P65, HO-1 and Nrf2 levels was examined in SK-N-MC cells. The proteins expression was normalized against β-actin. QCT down-regulates the P-IκBα and NF-κB P65, however it up-regulates the HO-1 and Nrf2 activity. Values were represented as mean ± SD from three independent experiments. Values were presented as mean ± SD. ^##^ *p* < 0.01, compared to the control group; * *p* < 0.01, ** *p* < 0.001, compared to the Mn alone group.

The NF-κB proteins are present in the cytoplasm as inactive heterodimers composed of two subunits, P50 and P65, and are bound to the inhibitory protein IκBα. The NF-κB (P-IκBα and NF-κB P65), HO-1 and Nrf2 protein levels were examined using western blotting method and the results were showed in Figure 4. Compared to control group, Mn exposure for 24 h significantly upregulated the levels of P-IκBα, NF-κB P65, HO-1 and Nrf2 proteins. QCT pretreatment markedly inhibited the level of P-IκBα and NF-κB P65, while further increased the level of HO-1 and Nrf2 (Figure 4).

### 2.5. The Beneficial Effect of QCT on Hematological Parameters against Mn-Induce Toxicity in Rat Brain

**Figure 5:**
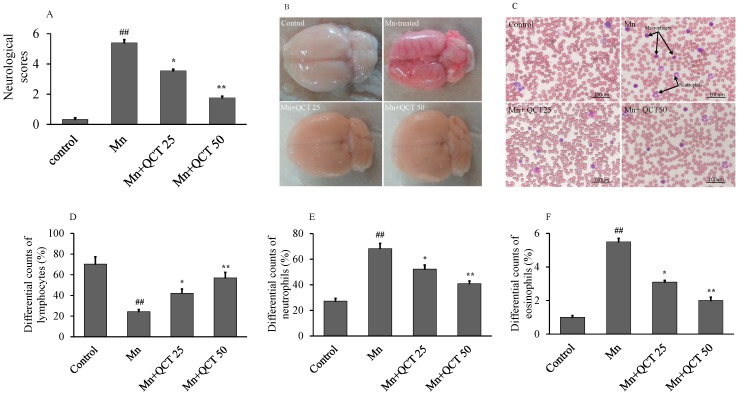
The beneficial effect of QCT on hematological parameters against Mn in rat brain. (**A**) Neurological scores; (**B**) effects of QCT on Mn-induced brain hemorrhage, scale bar: 100 µm; (**C**) microscopically view of neutrophils and macrophage (arrow), scale bar: 100 μm; (**D**–**F**) differential counts (%) of the lymphocytes, neutrophils and eosinophils. The number of blood cells was determined by calculating the percentage of differential blood cells over the 100 number of cells. Values were presented as mean ± SD. ^##^ *p* < 0.001 versus control group; * *p* < 0.01, ** *p* < 0.001 versus Mn group.

In comparison with the control group, neurological deficits were observed in the Mn group (5.63 ± 0.12 versus 0.33 ± 0.17, *p* < 0.001). In comparison with the Mn group, a significant difference was observed between the Mn + QCT 25 group (3.55 ± 0.19 versus 5.63 ± 0.12, *p* < 0.01) and the Mn + QCT 50 group (3.38 ± 0.81 versus 1.77 ± 0.25, *p* < 0.001 (Figure 5A). The brain hemorrhage with remarkable defects in the Mn group was observed, while QCT-treated both groups showed the beneficial effect (Figure 5B). In comparison with the control group, neutrophils and macrophage (arrows) were observed more in the Mn-treated group, where significantly decreased in the QCT-treated both groups (Figure 5C). Our results showed that compared with the control group, the Mn treatment significantly decreased (*p* < 0.001) the lymphocytes (24.40 ± 2.1 verses 70.4 ± 7.3, *p* < 0.001), while significantly increased (*p* < 0.001) the neutrophils (68.40 ± 4.1 verses 27.4 ± 2.3, *p* < 0.001) and the eosinophils (5.50 ± 0.2 verses 1 ± 0.1, *p* < 0.001). Interestingly, the treatment with QCT significantly reversed (*p* < 0.01 or *p* < 0.001) the Mn-induced changes in the lymphocytes, neutrophils and eosinophils (Figure 5D–F).

### 2.6. QCT Decreased ROS and Protein Carbonyl Levels and Restores Cu/Zn-SOD Activity on Mn-Induced Oxidative Damage in the Rat Brain

**Figure 6:**
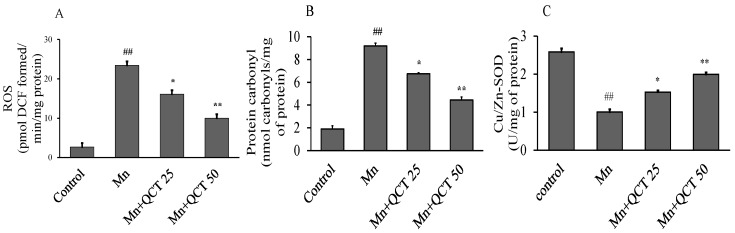
Effect of QCT on ROS and protein carbonyl levels and Cu/Zn-SOD activity in the Mn treated rat brain. Values are expressed as mean±SD. (**A**) Represents ROS levels; (**B**) represents protein carbonyl levels; (**C**) represents Cu/Zn-SOD enzymatic activity in rat brain. Values were presented as mean ± SD. ^##^ *p* < 0.001 versus control group; * *p* < 0.01, ** *p* < 0.001 versus Mn group.

To determine the protective effect of QCT against Mn-induced oxidative damage in rat brain, we examined the changes in the levels of ROS and protein carbonyl as well as the expression and activities of enzymes Cu/Zn-SOD. Mn treatment significantly increased (*p* < 0.001) ROS and protein carbonyl levels, while significantly decreased (*p* < 0.001) Cu/Zn-SOD activity in SD rat brain (Figure 6). Interestingly, QCT treatment effectively decreased (*p* < 0.01 or *p* < 0.001) ROS and protein carbonyl levels and increased (*p* < 0.01 or *p* < 0.001) Cu/Zn-SOD activity induced in Mn-treated groups.

### 2.7. QCT Decreases the Expression of Inflammatory Markers against Mn-Induced Neuroinflammation in the Rat Brain

**Figure 7:**
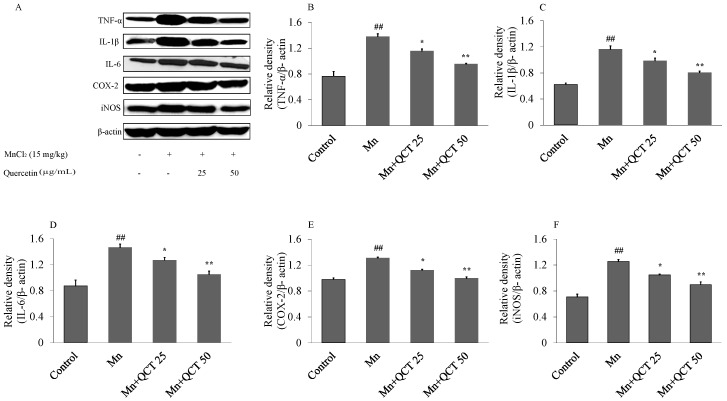
QCT decreases the expression of inflammatory markers in the brain of Rats. Values are expressed as mean ± SD. (**A**) Representative immunoblots for TNF-α, IL-1β, IL-6, COX-2, iNOS and β-actin in all the treated groups; (**B**–**F**) relative density analysis of the TNF-α, IL-1β, IL-6, COX-2 and iNOS protein bands. The relative density is expressed as the ratio (TNF-α, IL-1β, IL-6, COX-2, and iNOS /β-actin). ^##^ *p* < 0.001 versus control group; * *p* < 0.01, ** *p* < 0.001 versus Mn group.

In comparison with control group, Mn treatment significantly increased TNF-α, IL-1β, IL-6, cyclooxygenase-2 (COX-2) and iNOS protein expression in rats brain (TNF-α, *p* < 0.001; IL-1β, *p* < 0.001; IL-6, *p* < 0.001; COX-2, *p* < 0.001, iNOS, *p* < 0.001) (Figure 7), whereas QCT significantly decreased TNF-α, IL-1β, IL-6, COX-2 and iNOS protein expression in the brain of rats (TNF-α: *p* < 0.01 or *p* < 0.001; IL-1β: *p* < 0.01 or *p* < 0.001; IL-6: *p* < 0.01 or *p* < 0.001; COX-2: *p* < 0.01 or *p* < 0.001, iNOS: *p* < 0.01 or *p* < 0.001, versus Mn group) and the expression of these inflammatory markers was restored to normal levels.

### 2.8. QCT Down-Regulates the NF-κB, iNOS mRNA Levels and Up-Regulates the Nrf2, HO-1 mRNA Levels in the Rat Brain

**Figure 8:**
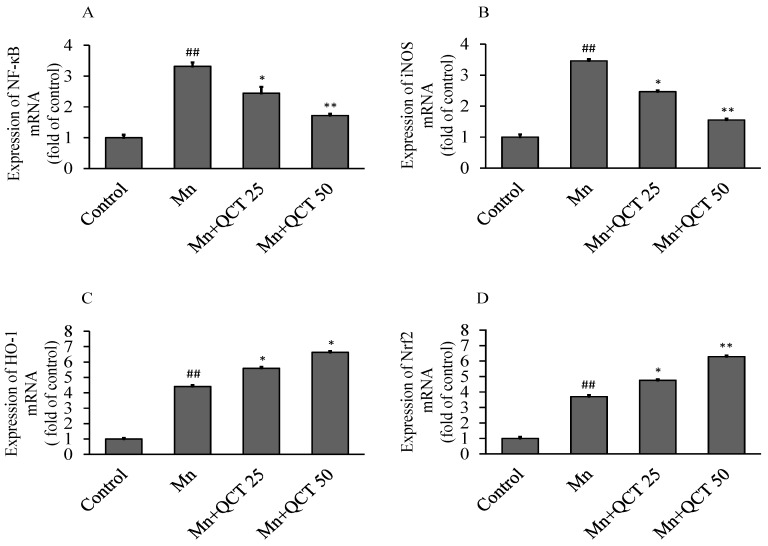
Effect of QCT on the expressions of NF-κB, iNOS, HO-1 and Nrf2 mRNA levels in rats brain. QCT treatment markedly inhibited the expression levels of NF-κB (**A**) and iNOS (**B**) mRNAs, but further increased the expression levels of HO-1(**C**) and Nrf2 (**D**) mRNAs. Values were presented as mean ± SD. ^##^ *p* < 0.01, compared to the control group; * *p* < 0.01, ** *p* < 0.001, compared to the Mn alone group.

The NF-κB, iNOS, HO-1 and Nrf2 RNA levels were examined using quantitative real time polymerase chain reaction (qRT-PCR) method and the results are showed in Figure 8. In comparison with control group, Mn treatment significantly (*p* < 0.001) upregulated the expression levels of NF-κB, iNOS, HO-1 and Nrf2 mRNA, to approximate 3.31, 3.24, 4.4 and 3.69-folds, respectively. QCT treatment markedly inhibited the expression level of NF-κB and iNOS mRNAs, while further increased the expression level of Nrf2 and HO-1 mRNAs.

### 2.9. QCT Decreased Neuroapoptosis Induced by Mn in the Rat Brain

**Figure 9:**
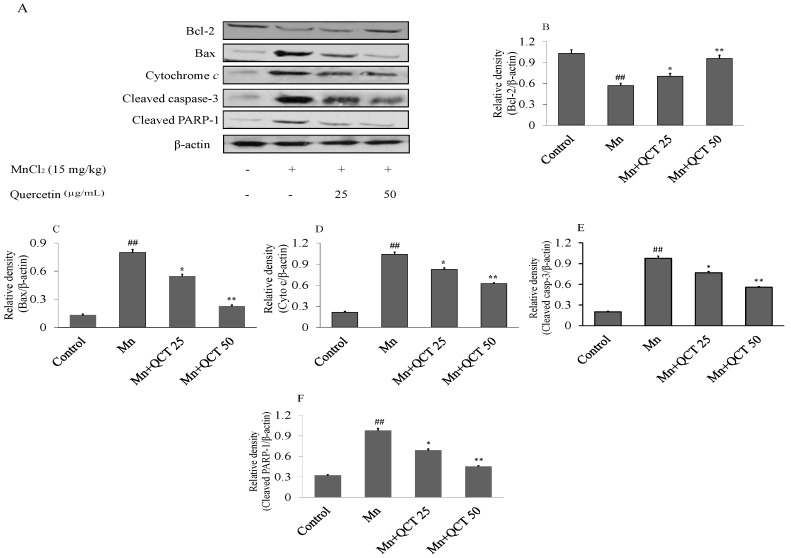
QCT reversed the apoptotic marker expression and neurodegeneration in the Mn-treated Rats. (**A**) The immunoblot analysis of the anti-apoptotic Bcl-2, pro-apoptotic Bax, cytochrome *c*, cleaved caspase-3, and the cleaved PARP-1 proteins in rat brain; (**B**–**F**) relative density analysis of the Bcl-2, Bax, cytochrome *c*, cleaved caspase-3, and the cleaved PARP-1 protein bands. The relative density is expressed as the ratio (Bcl-2, Bax, cytochrome *c*, cleaved caspase-3, and the cleaved PARP-1/β-actin). Values were presented as mean ± SD. ^##^ *p* < 0.001 versus control group; * *p* < 0.01, ** *p* < 0.001 versus Mn group.

Western blot analyses were performed to investigate the effects of Mn and QCT on the expression of Bcl-2, Bax, cytochrome *c*, cleaved caspase-3 and polymerase-1 (PARP-1) proteins in the rat brain (Figure 9). Our results showed that compared with the control treatment, the Mn treatment significantly decreased the expression of the anti-apoptotic protein Bcl-2, increased the expression of Bax, cytochrome *c*, cleaved caspase-3 and PARP-1 proteins. Interestingly, treatment with QCT significantly reversed the Mn-induced changes in Bcl-2, Bax, cytochrome *c*, cleaved caspase-3 and PARP-1 levels.

### 2.10. Effect of Treatment on Histopathological and Immunohistochemical Changes

**Figure 10:**
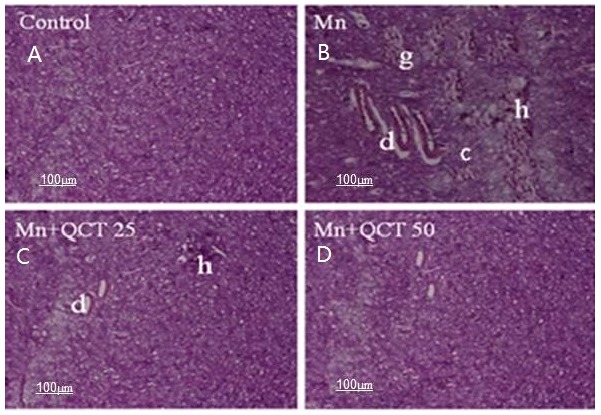
Photographs showing histopathological changes in striatum in different groups. (**A**) Control group (Normal saline); (**B**) MnCl_2_ (15 mg/kg) treated group; (**C**) MnCl_2_ (15 mg/kg) + QCT (25 mg/kg); (**D**) MnCl_2_ (15 mg/kg) + QCT (50 mg/kg) treated group. Damage (d), Ghost cells (g), hemorrhage (h), and vacuolated cytoplasm (c). Scale bar: 100 μm.

The histology of the striatum was examined under microscope (Figure 10). Exposure of Mn lead to marked histopathological alterations in the striatum, including increased neuronal loss, ghost cells, hemorrhage and vacuolated cytoplasm. No histopathological change was observed in striatum of normal control, while QCT treated groups showed beneficial effect.

**Figure 11:**
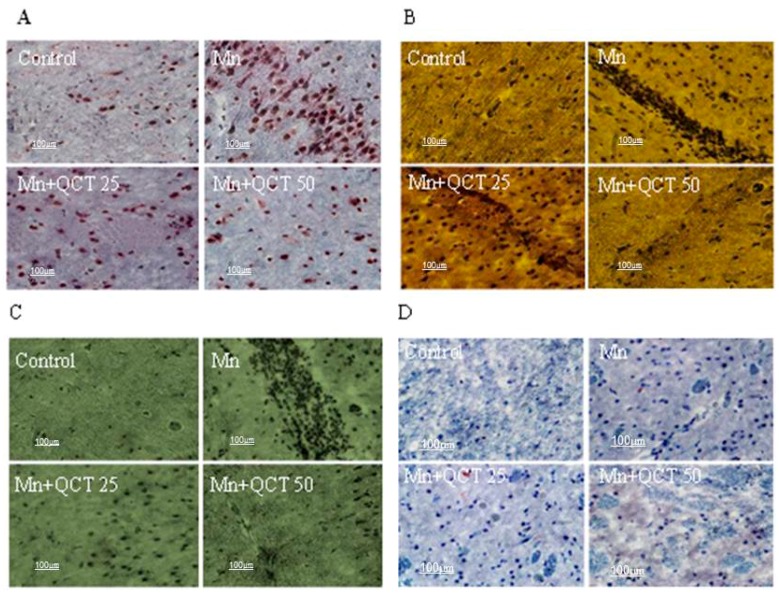
Immunohistochemistry (IHC) of QCT treatment along with Mn in striatum of rats under different treatment condition. (**A**) The 8-OHdG was examined in the striatum region; (**B**) the Bax was examined in the striatum region; (**C**) the activated caspase-3 was examined in the striatum region; (**D**) the activated PARP-1 was examined in the striatum region. Scale bar: 100 μm.

8-Hydroxy-2′-deoxyguanosine (8-OHdG), Bax, activated caspase-3 and PARP-1 immunoreactivity was significantly increased in microsection of rats treated with Mn when compared to control group (Figure 11). Moreover, immunoreactivity was significantly inhibited in groups treated with QCT (Figure 11).

## 3. Discussion

In the present study, we investigated the protective role of QCT on Mn-induced neurotoxicity and the potential mechanism in neuroblastoma SK-N-MC cells and SD male rats.

We determined the beneficial effects of QCT against the Mn-induced cytotoxicity by conducting cell viability and LDH release assays in SK-N-MC cells. Our results showed that Mn exposure at 500 μM significantly decreased cell viability (Figure 1A) and increased LDH activity (Figure 1B) in SK-N-MC cells after 24 h. However, pretreatment with QCT significantly attenuated Mn-induced alteration of cell viability and LDH activity, which could protect the SK-N-MC cell from cytotoxicity [36].

To understand the antioxidant effects of QCT against the Mn-induced oxidative stress, we quantified the intracellular ROS, MDA and GSH level and SOD and CAT activities in vitro (Figure 2). We found that Mn treatment resulted in a significant increase in the ROS and MDA levels, while significantly decreases GSH levels, SOD and CAT activities compared with the control treatment and QCT alone treatment, while QCT applied to Mn treated cells reverted these events suggesting QCT acted as a potent antioxidant against oxidative stress [36,37].

The main target of ROS-induced oxidative damage is mitochondria. In the present study, Mn-treatment significantly decreased ΔΨm of SK-N-MC cells (Figure 3A), which was an indicative character of mitochondrial dysfunction. However, QCT pretreatment effectively attenuated) the disruption of ΔΨm which was the integrators of intrinsic and extrinsic apoptotic signals and plays an important role in initiating mitochondria mediated apoptosis [37]. There are several pathways involved in the apoptosis process, including the mitochondrial pathway, death receptor pathway, and ER pathway [38,39]. Our previous study demonstrated Mn could induce the cell apoptosis in SK-N-MC cell line via the involvement of ER stress and mitochondria dysfunction [19]. In the present study, we found that Mn exposure significantly caused chromatin condensation, nucleus condenses, and formation of apoptotic bodies in SK-N-MC cells and finally lead to significant increase of the apoptotic rates [38]. Interestingly, pretreatment of QCT attenuated Mn-induced cytotoxicity involving the inhibition of cell apoptosis (Figure 3B,C).

In this study, we examined the protective effect of QCT against Mn-induced oxidative stress and neuroinflammation in an experimental rat model. Our data showed that, QCT improved neurobehavioral deficits (Figure 5A) and possessed the beneficial effect on hemorrhage (Figure 5B) and hematological parameters against Mn-induced toxicity in SD rats (Figure 5C–F) [27,40,41].

We examined the changes in the levels of ROS and protein carbonyl as well as the expression and activities of enzymes Cu/Zn-SOD to assess the effect of QCT against Mn-induced oxidative damage in rat brain. Our result demonstrated that, Mn treatment significantly elevated ROS and protein carbonyl levels and decreased Cu/Zn-SOD activity in SD rat brain. However, QCT-treated groups showed strong protection against Mn-induced alteration of ROS, protein carbonyl and Cu/Zn-SOD (Figure 6) [42,43].

Our results showed that Mn exposure resulted in the release of proinflammatory cytokines (TNF-α, IL-1β and IL-6) and the induction of enzymes (iNOS and COX-2) caused by activation of NF-κB (Figure 7). As a result, these changes may lead to brain inflammation and neurotoxic reactive nitrogen species (RNS) generation [44]. Interestingly, when the activation of NF-κB was inhibited by QCT, the inflammatory response was attenuated in the Mn + QCT groups (Figure 7).

NF-κB protein consists of P50 and P65 subunits, and κBα protein bound with them to prevent from the translocation into the nucleus of the cell [45]. 26S proteasome mediated pathway involved in phosphorylation and proteolytic degradation of IκBα that lead to translocation of NF-κB into the nucleus and regulates gene transcription [46]. The western blot analysis of NF-κB p65 was done to investigate whether QCT could affect nuclear translocation of NF-κB in SK-N-MC cell line. The NF-κB p65 protein expression was markedly increased in Mn alone group, while QCT significantly attenuated it (Figure 4). We also investigate the mode of translocation of NF-κB by analyzing phosphorylation of IκBα in cytoplasm using western blot analysis. We found that QCT significantly attenuated IκBα phosphorylation in SK-N-MC cells (Figure 4). Consistently, in the present the study, Mn exposure could significantly increase the expressions of NF-κB mRNA and iNOS mRNA that could attenuate with QCT pretreatment in SD rats (Figure 8A,B). Nrf2 is a critical transcription factor regulating the anti-oxidant genes such as GSH, CAT, SOD and HO-1 by binding to antioxidant response elements [47]. It has been reported that the upregulation of these enzymes due to Nrf2 transcription contributes to the cytoprotective adaptive response and plays an important role in response to oxidative stress [48]. In this study, we found that Mn exposure significantly increased the expression levels of HO-1 and Nrf2 mRNA gens (Figure 8C,D) and protein expressions (Figure 4). Consistently, QCT pretreatment further activated the Nrf2 and HO-1 mRNA (Figure 10C,D) and protein expressions (Figure 4).

Oxidative stress causes the depolarization of mitochondrial membrane, resulting in release of cytochrome *c* from the mitochondria into the cytosol, followed by the activation of caspase pathway, thus leading to apoptosis [49]. The activation of the pro-apoptotic protein Bax involved the intrinsic mitochondrial apoptotic pathway, resulting in the release of cytochrome c from the mitochondria into the cytosol, followed by the activation of caspase-9 and caspase-3, subsequently the activation of PARP-1 protein [50,51]. Our result showed that QCT treatment significantly prevented the Mn-induced neurodegeneration by attenuating various apoptotic markers (Figure 9).

**Figure 12:**
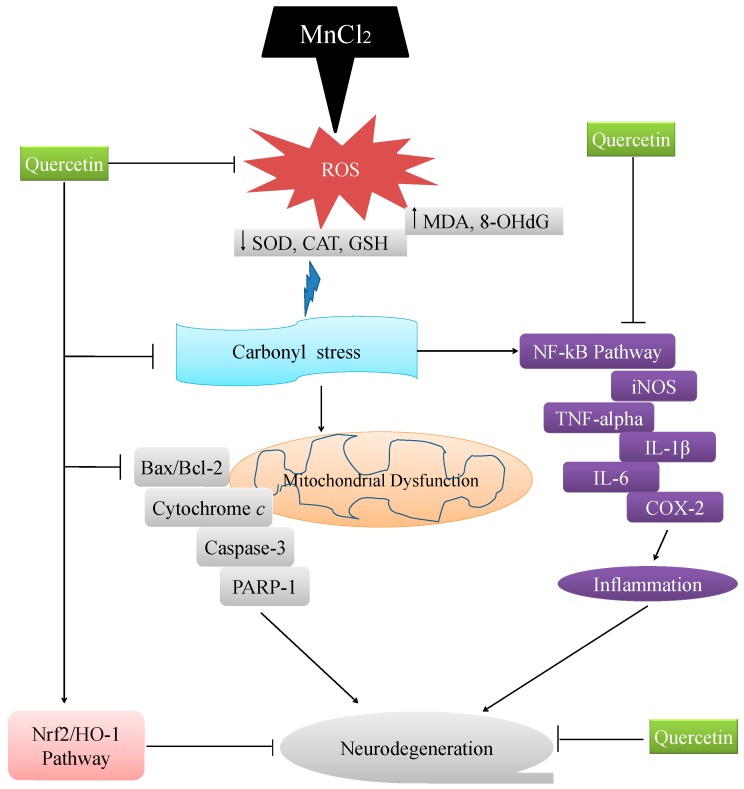
The proposed mechanism of action of QCT against the Mn-induced neurotoxicity. The schematic diagram showing the proposed mechanism underlying neuroprotective effect of QCT against Mn-induced ROS production, carbonyl stress, neuroinflammation and apoptotic neurodegeneration. This diagram shows that QCT prevents the Mn-induced ROS production, carbonyl stress, neuroinflammation and neurodegeneration by enhancing antioxidant activity through regulation of apoptosis, iNOS/NF-κB and HO-1/Nrf2 pathways. Arrow (→) lines indicate a stimulatory effect and T-bar indicates inhibitory effect.

We investigated the protective effects of QCT on Mn-induced neuronal toxicity in the striatum of SD male rats treated with MnCl_2_ at a dose regimen of 15 mg/kg. The striatum was chosen because Mn affects the striatum regions more severely than any other area of the CNS [41]. Exposure of Mn caused histopathological alterations in the striatum (Figure 12). Administration of QCT (25 and 50 mg/kg) to Mn-exposed rats showed improvement of histopathological alteration in comparison to Mn treated rats (Figure 10).

Oxidative stress causes neurological disorders such as Alzheimer’s disease (AD) and PD which are associated to 8-OHdG, produced during oxidation of DNA bases [52]. Our result revealed that 8-OHdG immunoreactivity was significantly increased in striatum region of Mn-exposed rats compared to normal control rats, whereas 8-OHdG immunoreactivity was markedly attenuated in QCT treated rats. Caspase upregulation and significant reduction of Bax immunoreactivity indicates alterations of neuron in the vulnerable brain regions of AD patients [53,54]. Administration of QCT to Mn-exposed rats showed significant reduction of activated caspase-3 and PARP-1 immunoreactivity (Figure 11).

## 4. Materials and Methods

### 4.1. Cell Viability

Crystal violet assay was used to determine Mn-induced cell death. Briefly, SK-N-MC cells were seeded in 24-well plates with 5 × 10^4^ cells per well in culture media and allowed to attach overnight. The cells were pretreated with the doses of QCT at 5, 10 and 20 µg/mL at 37 °C in a humidified atmosphere of 5% CO2/95% air for 6 h followed by the incubation with 500 μM Mn for 24 h. After 24 h of incubation, removed medium and washed the cells with phosphate buffer solution (PBS) and added 0.2% crystal violet solution to each well. After 10 min of incubation, the crystal violet solution was removed carefully by washing with water. Finally, added 100 μL 1% sodium dodecyl sulfate (SDS) to solubilize the color solution until the color is uniform and no areas of dense coloration in bottom of wells. The samples were read at 590 nm in a microplate reader (Spectra MAX, Gemini EM, Molecular Device, Sunnyvale, CA, USA). The cell viability is expressed as the relative abundance of viable cells.

### 4.2. Lactate Dehydrogenase (LDH) Activity

LDH release into the media was taken as an indicator of cell damage and the assay is based on the principle of reduction of nicotinamide adenine dinucleotide (NAD) by LDH. The reduced NAD (NADH) is utilized in the stoichiometric conversion of a tetrazolium dye which is measured spectrophotometrically using an assay kit Tox-7 (Sigma, Saint Louis, MO, USA). Briefly, SK-N-MC cells were seeded (5 × 10^4^ cells/well) and cultured in 24-well culture plates. The cells were then preincubated with or without different concentrations of QCT (5, 10, and 20 μg/mL) at 37 °C for 6 h followed by incubation with 500 μM MnCl_2_ (CAS: 7773-01-5, Sigma, Saint Louis, MO, USA) for 24 h. After treatment was over, cells were centrifuged at 240× *g* for 4 min and culture supernatant was transferred in a new plate. The assay mixture was prepared and added to each well and the plate incubated wrapped in foil at room temperature for 30 min. Reaction was terminated by adding the stop solution to each well. The plate was read at 490 nm at a reference wavelength of 690 nm. The extent of LDH leakage is expressed as the fold of absorbance of control.

### 4.3. Measurement of Intracellular Reactive Oxygen Species (ROS) Level

ROS level was measured by using 2′,7′-dichloro-dihydro-fluorescein diacetate (DCFH-DA) method [55]. DCFH-DA is a non-fluorescent compound, and it can be enzymatically converted to a highly fluorescent compound, DCF, in the presence of ROS. In brief, after the treatment, SK-N-MC cells were washed with PBS and incubated with DCFH-DA at a final concentration of 10 μmol/L for 30 min at 37 °C in darkness. The fluorescence intensity was measured in the microplate reader (Spectra MAX, Gemini EM, Molecular Device, Sunnyvale, CA, USA) at an excitation wave length of 485 nm and an emission wave length of 538 nm after the cells were washed three times with PBS to remove the extracellular DCFH-DA. To avoid photo-oxidation of DCF, the fluorescence image was collected by a single rapid scan (4-line average, total scan time of 4.33 s). The level of intracellular ROS was showed as a percentage of non-treated control.

### 4.4. Antioxidant Status

Antioxidant status of QCT was examined by measurement of intracellular malondialdehyde (MDA), superoxide dismutase (SOD), catalase (CAT), and glutathione (GSH). The activities of SOD and CAT and the levels of MDA and GSH were measured using specific assay kits according to the manufacturer’s instructions (Nanjing Jiancheng Co., Ltd., Nanjing, China). In brief, SK-N-MC cells were plated into 12-well plates at a density of 2 × 10^5^ cells per well and pre-treated with QCT (5, 10 or 20 µg/mL) at 37 °C for 6 h. After removing the medium containing QCT, the cells were incubated with or without Mn (500 µM) for 24 h. Cells were washed with cold PBS and lysed using the cell lysis buffer provided by the manufacturer. The cell lysates were centrifuged at 14,000× *g* for 10 min at 4 °C. Supernatants were collected and assayed for the levels of MDA and GSH and the activities of SOD and CAT. Protein concentrations were quantified using the BCA protein assay kit (Intron Biotechnology, Inc., Gyeonggi, Korea).

### 4.5. Measurement of Mitochondrial Membrane Potential (ΔΨm)

Harvested SK-N-MC cells the day before the experiment and seed 6-well plate with 2 × 10^5^ cells per well in culture media and allowed to attach overnight. The cells were pretreated with the doses of QCT at 5, 10 and 20 µg/mL at 37 °C for 6 h; the cells were washed and treated with Mn at the final concentration of 500 µM for additional 24 h. In the control group, cells were treated with QCT at 5, 10 and 20 µg/mL for 6 h, then replaced with the fresh medium containing 0.1% dimethyl sulfoxide (DMSO) for additional 24 h. finally, the seeded cells on the 6-well plate PBS were washed once, then incubated with 5,5′,6,6′-Tetrachloro-1,1′,3,3′-tetraethylbenzimidazolylcarbocyanine iodide (JC-1) for 30 min at 37 °C in the dark, including blank wells (with non-stained cells). After incubation, the cells were washed twice with PBS and visualized using an inverted fluorescence microscopy. Monomeric JC-1 green fluorescence emission and aggregate were measured at excitation wavelength 488 nm, emission wavelength 530 nm on a microplate reader (Spectra MAX, Gemini EM, Molecular Device, Sunnyvale, CA, USA).

### 4.6. Apoptosis Assay 

Hoechst33342 staining was conducted to distinguish apoptotic cells from normal cells. Briefly, SK-N-MC cells were seeded in 6-well plates with 2 × 10^5^cells per well in culture media and allowed to attach overnight. The cells were pretreated with the doses of QCT at 5, 10 and 20 µg/mL at 37 °C for 6 h followed by the incubation with 500 μM Mn for 24 h. After incubation, wash the cells seeded on the 6-well plate PBS once, then incubated with 5 μg/mL Hoechst 33342 for 15 min. Finally, washed twice with PBS and observed by inverted fluorescence microscopy (Axioskop 2 plus microscope, Carl Zeiss, Oberkochen, Germany). The apoptotic nuclei were counted from five non overlapping fields and expressed as a percentage of the total number of nuclei counted.

### 4.7. Experimental Animal and Treatments

Animal study was conducted using Sprague-Dawley (SD) male rats of seven week, weighing 220–250 g each were purchased from Damool Science (Daejeon, Korea). All rats were conditioned at 25 ± 2 °C and 45–55% relative humidity with 12 h light/dark cycle and kept in clean and dry polypropylene cages. A standard laboratory diet and water ad libitum were provided. After acclimatization, the animals were randomly divided into four groups. The study was approved by Institutional Animal Care and Usage Committee (IACUC) of Chonbuk National University, Republic of Korea (Approved number: CBNU 2016-45; 1 July 2016).

Details of the experimental design are described in the supporting information, supplementary materials and methods.

### 4.8. Neurological Scoring

Details of the Neurological scoring are described in the supporting information, supplementary materials and methods.

### 4.9. Preparation of Peripheral Blood Smears and Differential Counts of WBC

Details of the preparation of peripheral blood smears and Differential counts of WBC are described in the supporting information, supplementary materials and methods.

### 4.10. Tissue Homogenates

Animals were deeply anaesthetized and sacrificed. Brains were promptly dissected and perfused with ice-cold 50 mM (pH 7.4) PBS. Details of the methods for tissue homogenates are described in the supporting information, supplementary materials and methods.

### 4.11. Collection of Brain Slices

Details are given in the supporting information, supplementary materials and methods. Coronal sections (12 μm) from cryofixed tissue were collected on 3-aminopropyl-trimethoxysilane-coated slides (Sigma-Aldrich, St. Louis, MO, USA) and stored at −70 °C. Details of the collection of brain are described in the supporting information, supplementary materials and methods.

### 4.12. Assay of ROS

Measurement of ROS was based on the oxidation of DCFH-DA to 2′7′-dichlorofluorescein, previously described method. Details of the assay of ROS are described in the supporting information, supplementary materials and methods.

### 4.13. Protein Carbonyls Assay

Protein carbonyl content was determined as a marker of oxidative damage to proteins. Details of the protein carbonyls assay are described in the supporting information, supplementary materials and methods.

### 4.14. Assay of Cu/Zn-SOD Activity

Chemicals used in the assay, including xanthine, xanthine oxidase, cytochrome c, bovine serum albumin (BSA) and SOD, were purchased from Sigma-Aldrich. Cu/Zn-SOD activity was measured according to previously described method. Details of the assay of Cu/Zn-SOD activity are described in the supporting information, supplementary materials and methods.

### 4.15. Quantitative Real Time Polymerase Chain Reaction (qRT-PCR)

In order to investigate the protective mechanism of QCT, the expression of NF-κB, iNOS, HO-1 and Nrf-2 were examined by quantitative real time polymerase chain reaction (qRT-PCR). Total RNA was extracted from rat brain using Trizol reagent (sigma-Aldrich, Saint Louis, MO, USA). Details are given in the supporting information, supplementary materials and methods.

### 4.16. Western Blot Analysis

Proteins extracted from either brain tissues (80 µg) or SK-N-MC cells (40 µg) were analyzed by Western blotting. Details are given in the supporting information, supplementary materials and methods.

### 4.17. Histology and Immunohistochemistry

Histological evaluation was performed following the method of Wang et al. [41], on the striatum of brains from control and experimental groups after embedded in optimal cutting temperature (OCT, Leica Biosystems Melbourne Pty Ltd., DB Maarn, The Netherland) medium. The pathological changes were viewed under light microscope after staining with hematoxylin and eosin. In order to determine the effect of the treatments on the reactivity of oxidative protein 8-OHdG, pro-apoptotic protein Bax, apoptotic protein caspase-3 and PARP-1 immunohistochemistry was performed in the striatum of all experimental groups, respectively. Details are given in the supporting information, supplementary materials and methods.

### 4.18. Statistical Data Analysis 

All the data were expressed as mean ± SD and one way ANOVA (Analysis of variance) followed by Dunnett’s test was used for the statistical analysis using SPSS software (version 16, SPSS, Inc., Chicago, IL, USA). * *p* < 0.01 and ** *p* < 0.001 were considered significant.

## 5. Conclusions

In conclusion, the present study reveals that carbonyl stress, mitochondrial apoptotic pathway and inflammatory response participate in Mn-induced neurotoxicity (Figure 12). Importantly, QCT could effectively attenuate the Mn-induced ROS production, carbonyl stress, neuroinflammation and neurodegeneration by enhancing antioxidant activity through regulation of apoptosis, iNOS/NF-κB and HO-1/Nrf2 pathways (Figure 12).

## Supplementary Materials

Supplementary materials can be found at www.mdpi.com/1422-0067/18/9/1989/s1.

## Abbreviations

Mn	Manganese
QCT	Quercetin
LDH	Lactate dehydrogenase
WBC	White blood cell
h	Hour
ER	Endoplasmic reticulum
PD	Parkinson’s disease
AD	Alzheimer’s disease
ROS	Reactive oxygen species
MDA	Malondialdehyde
SOD	Superoxide dismutase
CAT	Catalase
GSH	Glutathione
HO-1	Heme oxygenase 1
WBC	White blood cell
8-OHdG	8-Hydroxy-2′-deoxyguanosine
TNF-α	Tumor necrosis factor-α
IL-1β	Interleukin-1β
IL-6	Interleukin-6
COX-2	Cyclooxygenase-2
iNOS	Inducible nitric oxide synthase
SD	Sprague-dawley
PBS	Phosphate buffer solution
DCFH-DA	2′,7′-dichloro-dihydro-fluorescein diacetate
qRT-PCR	Quantitative real time polymerase chain reaction
OCT	Optimal cutting temperature
