# Supplementary material for: Quercetin Attenuates Manganese-Induced Neuroinflammation by Alleviating Oxidative Stress through Regulation of Apoptosis, iNOS/NF-κB and HO-1/Nrf2 Pathways
Source: Int J Mol Sci. 2017 Sep 15;18(9):1989. doi: 10.3390/ijms18091989 (PMC5618638; doi:10.3390/ijms18091989)
Supplement: Supplementary file 1 [file ijms-18-01989-s001.pdf]

Supplementary Materials: Quercetin attenuates manganese-induced neuroinflammation by alleviating oxidative stress through regulation of apoptosis, iNOS/NF-κB and HO-1/Nrf2 pathways

Entaz Bahar [1], Ji-Ye Kim [2, 3], and Hyonok Yoon [1,*]

[1] College of Pharmacy, Research Institute of Pharmaceutical Sciences, Gyeongsang National University, Jinju 52828, Gyeongnam, Republic of Korea; entaz_bahar@yahoo.com

[2] Department of Pathology, College of Medicine, Yonsei University, Seoul 03722, Republic of Korea; alucion@gmail.com

[3] Department of Pathology and Translational Genomics, School of Medicine, Samsung Medical Center, Seoul 06351, Republic of Korea, alucion@gmail.com

*Correspondence: hoyoon@gnu.ac.kr; +82-55-772-2422; Fax: +82-55-772-2409

Supplementary Materials and Methods

1. Experimental Animal and Treatments

Seven-week-old Sprague-Dawley (SD) male rats, weighing 220 - 250 g each were purchased from Damool Science (Daejeon, Republic of Korea). They were kept in clean and dry polypropylene cages with 12 h light, dark cycle at 25 ± 2 °C and 45 – 55 % relative humidity in the animal house, Pharmacology department, Chonbuk national university. The rats were fed with a standard laboratory diet and water ad libitum. After a week of adaptation, the rats were randomly divided into four groups. The protocol used for this study in the rat as an animal model was carried out with the guidelines of the Institutional Animal Care and Usage Committee (IACUC) and approval was taken from ethical committee of Chonbuk National University, Korea for using animals by describing the protocols of the study (Approved number: CBNU 2016-45).

The general condition of the animals was checked daily. The animals were acclimated for 7 days before the beginning of the experiment. Thereafter, they were randomly distributed into 4 groups and a sub-chronic in vivo assay was performed according to the following protocol: group 1, the control group was intraperitoneal (IP) injected with normal saline solution (for 8 days, every 24 h); group 2, administration of 8 doses of $MnCl_2$ (15 mg/kg) by IP injection, every 24 h (for 8 days); group 3, administration of 8 doses of $MnCl_2$ (15 mg/kg) by IP injection after orally (PO) giving QCT (25 mg/kg) every 24 h (for 8 days); group 4, administration of 8 doses of $MnCl_2$ (15 mg/kg) by IP injection after PO giving QCT (50 mg/kg) every 24 h (for 8 days) (see Table 1 and Fig.S1).

Table S1: Experimental design

Groups*	Treatment solution	Administration
Control	Normal saline (IP)	8 doses
Mn	15mg $MnCl_2$/kg (IP)	8 doses
Mn + QCT	15mg $MnCl_2$ (IP) +25 mg/kg QCT (PO)	8 doses+8 doses
Mn + QCT	15mg $MnCl_2$ (IP) +50 mg/kg QCT (PO)	8 doses+8 doses

*Animals that died during treatments were not allocated to any group. Upon death, animal subjects were replaced until the final group size reached the expected number, resulting in an equal number of subjects in each group. All treatment solutions were prepared freshly on a daily basis.

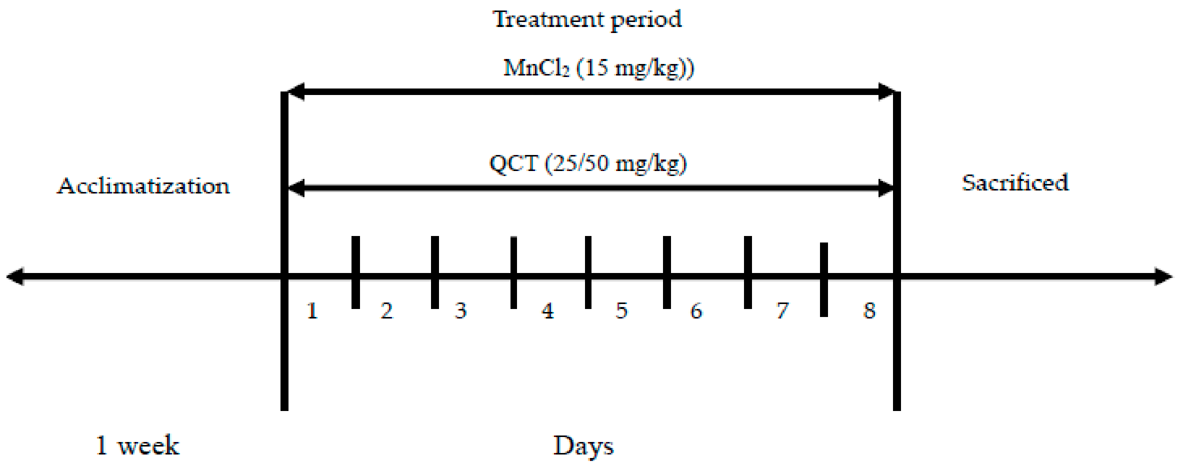

Figure S1: Treatment patterns

2. Neurological Scoring

The neurological function of all animals was recorded during treatment periods using a previously described scoring system for rodents [1, 2]. The behavior and activity scores were as follows: 1, appetite (finished meal 0; left meal unfinished 1; scarcely ate 2); 2, activity (walked and reaches at least three corners of the cage 0; walked with some stimulation 1; almost always lying down 2); and 3, deficits (no deficits 0; unstable walk 1; impossible to walk 2). The minimum

neurological score is 0 and the maximum is 6, which means that neurological deficits were graded as a total score from 0 to 6. A higher score represents more serious neurological deficits. The scoring was conducted by two independent researchers who were blind to treated groups.

Table S2: Neurological score

Categories	Behaviors and activities*	Score
	Finished meal	0
**Appetite**	Left meal unfinished	1
	Scarcely ate	2
	Active, barking, or standing	0
**Activity**	Lying down, will stand and walk with some stimulation	1
	Almost always lying down	2
	No deficits	0
**Deficits**	Unstable walk due to ataxia or paresis	1
	Impossible to walk and stand because of ataxia or paresis	2

*Behavioral examinations were performed daily after treatment to record appetite, activity, and neurological deficits.

3. Preparation of Peripheral Blood Smears and Differential Counts of WBC

A blood film or peripheral blood smear is a thin layer of blood smeared on a glass microscope slide and then stained in such a way as to allow the various blood cells to be examined microscopically. Blood films were made by placing a drop of blood on one end of a slide, and using a spreader slide to disperse the blood over the slide's length. The slides were left to air dry, after which the blood was fixed to the slide by immersing it briefly in methanol. After fixation, the slide is stained to distinguish the cells from each other. Diff-Quik, commercial Romanowsky stain variant was used to rapidly stain and differentiate a variety of smears, commonly blood and non-gynecological smears, including those of fine needle aspirates [3-5]. Briefly, Dipped peripheral blood smears slide into fixative reagent (Triarylmethane dye and methanol), then dipped slide into stain solution 1 (Eosin G in phosphate buffer) followed by stain solution 2 (Thiazine dye in phosphate buffer) and allow excess

to drain after each dip. Rinsed slide in distilled water (P^H 7.2) and allow dry in air. Then visualized under Nikon ECLIPSE E600 microscope. The blood cells were counted in at least 200 cells from five non overlapping fields in all treatment, and expressed as a percentage of the total number of cells counted.

4. Tissue Homogenates

For biochemical studies performed as described previously [6]. Animals were sacrificed by decapitation. Brains were promptly dissected and perfused with ice-cold 50 mM (p^H 7.4) PBS. Brains were homogenized in 1/4 (w/v) ice-cold RIPA lysis buffer (1× Tris buffered saline (TBS) containing 30 μL of protease inhibitors cocktail (Sigma-Aldrich, MO, USA) per gram of tissue with 10 strokes at 1200 rpm in a Potter homogenizer. Homogenates were divided into three portions in which one part was centrifuged at 13000 × g for 10 min at 4 °C to obtain the supernatant and supernatant aliquots were used to determine brain ROS and protein carbonyl levels. The second part was sonicated four times for 30 s with 20 s intervals using a Bronson Scientific sonicator and centrifuged at 5000 × g for 10 min at 4 °C, and the supernatants were collected for determination of copper-zinc SOD (Cu/Zn-SOD) enzyme activities. The third part was sonicated four times for 30 s with 20 s intervals using a Bronson Scientific sonicator, centrifuged at 15,000g for 10 min at 4 °C, and the supernatant was collected and stored at −70 °C for Western blot studies. Protein levels in the supernatants were determined using the BCA assay kit (Intron Biotechnology, Inc., Gyeonggi, Republic of Korea).

5. Assay of ROS

Measurement of ROS was based on the oxidation of 2′7′-dichlorodihydrofluorescein diacetate to 2′7′-dichlorofluorescein [7, 8]. Briefly, the homogenate was diluted 1:20 times with ice-cold Locke's buffer to obtain a concentration of 10 mg tissue/ml. The homogenates were then pipetted into 24-well plates (1 mL/well) and allowed to warm to room temperature (21 ºC) for 5 min. At that time, 10 μl of DCFH-DA (10 μM final concentration) was added to reaction mixture containing Locke's buffer (p^H 7.4) with 0.2 mL homogenate and the plates preincubated for 15 min at room temperature to allow the DCFH-DA to be incorporated into any membrane-bound vesicles and the diacetate group cleaved by esterases. After 30 min, the conversion of DCFH to the fluorescent product DCF was measured using a microplate reader (Spectra MAX, Gemini EM, Molecular Device) with excitation at 485 nm and emission at 530 nm. Background fluorescence (conversion of DCFH-DA in the absence of homogenate) was corrected by the inclusion of parallel blanks. ROS formation was quantified from a DCF-standard curve and data are expressed as pmol DCF formed/min/mg protein. Protein

concentration in homogenates was determined using a commercial kit using BCA kit (Intron Biotechnology, Inc., Gyeonggi, Republic of Korea).

6. Protein Carbonyls Assay

Protein carbonyl content was determined as a marker of oxidative damage to proteins [7]. They were determined in supernatants obtained after centrifugation of tissue homogenates at 10,000 rpm for 15 min by measuring the hydrazone derivatives between 360 and 390 nm (e = 21.0 mM^{-1} cm^{-1}) using a microplate reader (Spectra MAX, Gemini EM, Molecular Device) according to the method described earlier [9]. Data are expressed as nmol carbonyls/mg protein.

7. Assay of Cu/Zn-SOD Activity

Chemicals used in the assay, including xanthine, xanthine oxidase, cytochrome c, bovine serum albumin (BSA) and SOD, were purchased from Sigma-Aldrich. Cu/Zn-SOD activity was measured according to a previously described method [10, 11]. Solution A was prepared by mixing 100 mL of 50-mM PBS (p^H 7.4) containing 0.1 mM ethylenediaminetetraacetic acid (EDTA) and 2 µmol of cytochrome c with 10 mL of 0.001 N NaOH solutions containing 5 µmol of xanthine. Solution B contained 0.2 U xanthine oxidase/mL and 0.1 mM EDTA. Fifty microliters of a tissue supernatant was mixed with 2.9 mL of solution A, and the reaction was started by adding 50 µL of solution B. Change in absorbance at 550 nm was monitored in a microplate spectrophotometer (Spectra MAX, Gemini EM, Molecular Device). A blank was run by replacing the supernatant with 50 µL of ultrapure water. Cu/Zn-SOD level was expressed as units per milligram protein, with reference to the activity of a standard curve of bovine Cu/Zn-SOD under the same conditions.

8. Western Blot Analysis

Proteins extracted from either brain tissues (80 µg) or SKNMC cells (40 µg) were analyzed by Western blotting. Briefly, total proteins were extracted and the protein concentration was determined using BCA kit (Intron Biotechnology, Inc., Gyeonggi, Korea). The protein samples were separated on desired % polyacrylamide gels and electro-transferred onto nitrocellulose (NC) membranes in a semi-dried environment. Blots were blocked by 5% defatted milk in Tris-buffer containing 0.1% Tween-20 and then incubated with the primary antibodies included: P-IκBα (SC-371, Santa Cruz Biotechnology, Inc.), NF-κB P65 (SC-372, Santa Cruz Biotechnology, Inc.), HO-1 (SC-10789, Santa Cruz Biotechnology, Inc.), Nrf2 (SC-722, Santa Cruz Biotechnology, Inc.), IL-1β (SC-7884, Santa Cruz Biotechnology, Inc.), IL-6 (SC-1265, Santa Cruz Biotechnology, Inc.), TNF-α (SC-1350, Santa Cruz Biotechnology, Inc.),

iNOS, COX-2 (SC-1746, Santa Cruz Biotechnology, Inc.), Bax (SC-23959, Santa Cruz Biotechnology, Inc.),, Bcl (SC-7382, Santa Cruz Biotechnology, Inc.), 2, cleaved caspase-3 (1:1,000, Asp175, 9661, cell signaling, Danvers, MA), Cytochrome-c (556432, BD Biosciences), PARP-1 (SC-1562, Santa Cruz Biotechnology, Inc.), and β-actin (A5441, Sigma-Aldrich, MO, USA) at 4 °C overnight. Subsequently, the blots were incubated with secondary antibodies included anti-mouse (#115-035-003; Jackson ImmunoResearch laboratories, Inc.), anti-goat (SC-2020 Santa Cruz Biotechnology, Inc.), and anti-rabbit (SC-2004, Santa Cruz Biotechnology, Inc.) at room temperature for 1 h. Then the blots were developed with EZ-Western Lumi Plus solution (ATTO Corporation, Tokyo, Japan) (Millipore Corporation, Billerica, MA, USA) and analyzed with Ez-Capture ST (ATTO Corporation).

9. Quantitative Real Time Polymerase Chain Reaction (qRT-PCR)

In order to investigate the protective mechanism of QCT, the expression of NF-κB, iNOS, HO-1 and Nrf-2 were examined by real time quantitative PCR. Total RNA was extracted from rat brain using Trizol reagent (sigma-Aldrich, Saint Louis, MO, USA). The RNA integrity was spectrophotometrically examined according to its A_{260}/A_{280} absorption. Subsequently, cDNA was synthesized through reverse transcription. Real time quantitative PCR was performed on Mastercycler ep realplex (Eppendorf, Hamburg, Germany). Briefly, the amplification reaction was carried out with 40 cycles at a melting temperature of 94 °C for 15 sec, an annealing temperature of 60 °C for 1 min, and an extension temperature of 72 °C for 50 sec. Housekeeping gene GAPDH was selected as an internal control. Following primers were used in the amplification: Nrf2: forward, 5′-ACA CGG TCC ACA GCT CAT C -3′, reverse, 5′- TGT CAA TCA AAT CCA TGT CCT G-3′; HO-1: forward, 5′-ACA GGT TGA CAG AAG AGG CTA A-3′, reverse, 5′- AAC AGG AAG CTG AGA GTG AGG -3′; NF-kB p6): forward, 5′-CTC CGC GGG CAG CAT CC-3′; reverse, 5′- AGC CGC ACA GCA TTC AGG TCG TAG -3′; iNOS: forward, 5′-CAG GAC CAC ACC CCC TAG GA-3′ , reverse, 5′- AGC CAC ATA CCG AGC CAT GC-3′; GAPDH: forward, , 5′-TGG AGT CTA CTG GCG TCT T-3, reverse, 5′- TGT CAT ATT TCT CGT GGT TCA -3′.The fold or percentage change in the relative expression of the target gene at the mRNA level was determined by the $2^{-\Delta\Delta Ct}$ method.

10. Collection of Brain Slices

The rats were deeply anesthetized with ketamine and perfused transcardially with 100 ml of normal saline (0.9 %). The brain tissues were fixed in a fresh solution of 4% paraformaldehyde (p^H 7.4) for 12 h, incubated overnight at 4 °C in 100 mM sodium phosphate buffer (p^H 7.4) containing 15 % sucrose followed by 30 % sucrose; and embedded in Optimal Cutting Temperature (OCT, Leica Biosystems Melbourne Pty Ltd., DB Maarn, Netherland). Coronal sections (10 μm) from cryofixed

tissue were collected on Silane-coated slides (Muto Pure Chemical Co., Ltd., South Korea) for histology and immunohistochemistry and stored at -70 °C.

11. Histology and Immunohistochemistry

Histological evaluation was performed following the method of Bahar et al., on the striatum of brains from control and experimental groups after embedded in Optimal cutting temperature (OCT) medium [19]. The pathological changes were viewed under light microscope after staining with hematoxylin and eosin.

In order to determine the effect of the treatments on the reactivity of oxidative protein 8-hydroxy-2' –deoxyguanosine (8-OHdG), pro-apoptotic protein Bax, apoptotic protein caspase-3and PARP-1 immunohistochemistry was performed in the striatum and cortex of all experimental groups, respectively. Sections were made following the same procedure as described above. The tissue were treated with mouse polyclonal anti-8-OHdG (1:500, N45.1, ab48508, Abcam) and rabbit polyclonal anti-Bax (1:500; P-19, sc-526, Santa Cruz Biotechnology, Inc.), rabbit polyclonal anti-caspase (1:500, Asp175, 9661, cell signaling, Danvers, MA) and mouse polyclonal anti-PARP-1(1:500, A-20, sc-1562, cell signaling Santa Cruz Biotechnology, Inc.) antibodies at 4 °C overnight. Subsequently, these were incubated with biotinylated goat anti-mouse (1:30, code: D 0314, Dako) and goat anti-rabbit (1:80, code: D 0487, Dako) immunoglobulins and latter visualized with substrate chromogen (Code: K3464, Dako), followed by hematoxylin and mounted with aqueous mount medium. The sections were dehydrated and cover slipped, viewed under a microscope, and photomicrographs were taken by Nikon Differential Interference Contrast inverted microscope (Nikon, Kanagawa, Japan) equipped with Narishige micromanipulators (Narishige, Tokyo, Japan).

12. Statistical Data Analysis

All the data were expressed as mean±SD and one way ANOVA (Analysis of variance) followed by Dunnett's test was used for the statistical analysis using SPSS software (version 16). $*p < 0.01$ and $**p < 0.001$ were considered significant.

Reference

1. Dong, Y. S.; Wang, J. L.; Feng, D. Y.; Qin, H. Z.; Wen, H.; Yin, Z. M.; Gao, G. D.; Li, C., Protective effect of quercetin against oxidative stress and brain edema in an experi mental rat model of subarachnoid hemorrhage. *International journal of medical sciences* **20 14,** 11, (3), 282-90.
2. Yamaguchi, M.; Zhou, C.; Nanda, A.; Zhang, J. H., Ras protein contributes to cerebral vasospasm in a canine double-hemorrhage model. *Stroke* **2004,** 35, (7), 1750-5.
3. Nakada, K.; Fujisawa, K.; Horiuchi, H.; Furusawa, S., Studies on morphology and cytoc

hemistry in blood cells of ayu Plecoglossus altivelis altivelis. *The Journal of veterinary medical science* **2014,** 76, (5), 693-704.
4. Woronzoff-Dashkoff, K. K., The wright-giemsa stain. Secrets revealed. *Clinics in laboratory medicine* **2002,** 22, (1), 15-23.
5. Horobin, R. W.; Walter, K. J., Understanding Romanowsky staining. I: The Romanowsky-Giemsa effect in blood smears. *Histochemistry* **1987,** 86, (3), 331-6.
6. Lu, J.; Wu, D. M.; Zheng, Y. L.; Sun, D. X.; Hu, B.; Shan, Q.; Zhang, Z. F.; Fan, S. H., Trace amounts of copper exacerbate beta amyloid-induced neurotoxicity in the cholesterol-fed mice through TNF-mediated inflammatory pathway. *Brain, behavior, and immunity* **2009,** 23, (2), 193-203.
7. Shinomol, G. K.; Muralidhara, Differential induction of oxidative impairments in brain regions of male mice following subchronic consumption of Khesari dhal (Lathyrus sativus) and detoxified Khesari dhal. *Neurotoxicology* **2007,** 28, (4), 798-806.
8. Driver, A. S.; Kodavanti, P. R.; Mundy, W. R., Age-related changes in reactive oxygen species production in rat brain homogenates. *Neurotoxicology and teratology* **2000,** 22, (2), 175-81.
9. Levine, R. L.; Garland, D.; Oliver, C. N.; Amici, A.; Climent, I.; Lenz, A. G.; Ahn, B. W.; Shaltiel, S.; Stadtman, E. R., Determination of carbonyl content in oxidatively modified proteins. *Methods in enzymology* **1990,** 186, 464-78.
10. Lu, J.; Zheng, Y. L.; Luo, L.; Wu, D. M.; Sun, D. X.; Feng, Y. J., Quercetin reverses D-galactose induced neurotoxicity in mouse brain. *Behavioural brain research* **2006,** 171, (2), 251-60.
11. Lu, J.; Wu, D. M.; Zheng, Y. L.; Hu, B.; Zhang, Z. F., Purple sweet potato color alleviates D-galactose-induced brain aging in old mice by promoting survival of neurons via PI3K pathway and inhibiting cytochrome C-mediated apoptosis. *Brain pathology* **2010,** 20, (3), 598-612.
